# OCCS Classification and Treatment Algorithm for Comminuted Mandibular Fractures Based on 109 Patients and 11 Years Experiences: A Retrospective Study

**DOI:** 10.3390/jcm11216301

**Published:** 2022-10-26

**Authors:** Xiaofeng Xu, Fangxing Zhu, Chengshuai Yang, Bing Xu, Zhaoqi Yuan, Wenbin Zhang, Jun Shi

**Affiliations:** 1 Department of Oral and Cranio-Maxillofacial Surgery, Shanghai Ninth People’s Hospital, Shanghai Jiao Tong University School of Medicine, Shanghai 200011, China; 2 College of Stomatology, Shanghai Jiao Tong University, Shanghai 200011, China; 3 National Center for Stomatology, Shanghai 200011, China; 4 National Clinical Research Center for Oral Diseases, Shanghai 200011, China; 5 Shanghai Key Laboratory of Stomatology, Shanghai 200011, China; 6 Shanghai Research Institute of Stomatology, Shanghai 200011, China; 7 Department of Oral and Maxillofacial-Head and Neck Oncology, Shanghai Ninth People’s Hospital, Shanghai Jiao Tong University School of Medicine, Shanghai 200011, China; 8 Department of Plastic and Reconstructive Surgery, Shanghai Ninth People’s Hospital, Shanghai Jiao Tong University School of Medicine, Shanghai 200011, China; 9 Shanghai Key Lab of Tissue Engineering, Shanghai Ninth People’s Hospital, Shanghai Jiao Tong University School of Medicine, Shanghai 200011, China

**Keywords:** comminuted mandible fracture, classification, titanium mesh, open reduction and internal fixation, staged operations

## Abstract

Comminuted mandibular fractures (CMFs) pose significant challenges to surgeons for their serious complications and poor outcomes. We aimed at proposing a classification with treatment algorithm of each category for CMFs. Patients with CMFs were retrospectively reviewed and classified into five categories: Type I: relatively good occlusion, no or slightly displaced fragments, no continuity destruction or bone defect; Type II: relatively good occlusion, damaged morphology, low comminution degree but intact continuity without bone defect; Type III: damaged morphology and higher comminution degree with intact continuity and relatively good occlusion; Type IV: high comminution, impaired continuity and poor occlusion without segmental bone defect; Type V: segmental bone defect. Conservative treatment, open reduction and internal fixation or microvascular osteocutaneous free flap transplantation was performed, accordingly. Demographics, perioperative data, complications and reasons for reoperations were recorded. The chi-square test was used for statistical analysis. In total, 109 patients were included in the study. After surgery, in the following group, 5 manifested infections, 1 manifested bone non-union, and 2 experienced reoperations, while in the unfollowing group, 10 manifested infections, 5 manifested bone non-union and 8 experienced reoperations. The OCCS classification and algorithm for CMFs achieve better outcomes and with lower complication rate.

## 1. Introduction

CMFs, defined as the presence of multiple fracture lines resulting in many small pieces of bone within the same area of mandible (at least 2 separated bone pieces) [1], account for only approximately 5–7% among maxillofacial trauma [2]. However, the treatment of CMFs is quite a challenge for most surgeons because of its serious postoperative complications, such as infection, malocclusion, facial deformity, bone non-union and even bone defects. The appearance and function of the patients may be affected severely by the aforementioned complications [2,3,4,5].

The recommendation of the Association for the Study of Internal Fixation (AO/ASIF) for CMFs is rigid fixation with reconstruction plates and bicortical screws. The use of MMF or miniplates fixation indiscriminately is not considered to produce enough stable osteosynthesis strength; therefore, it is incorrect and can often lead to postoperative complications [2]. Nevertheless, in actual scenarios, it is complicated and varies in clinical practice, and there is not one specific method that can cover the management of all kinds of CMFs. It has been reported that in severely comminuted or infected mandibles, fractures for which a staged internal fixation is planned and select pediatric or atrophic mandible fractures could be the indications of external fixation [6]. In years of clinical practice, the treatment philosophy has changed gradually and reached a consensus in our department after the year 2015. A systematic, standard treatment-oriented classification and treatment algorithm for CMFs were proposed. By following these, better treatment outcomes and a lower complication rate than before were received. Thus, we wish to share OCCS (**O**cclusal relationship, **C**ontinuous mandibular morphology, degree of mandibular **C**omminution and **S**egmental mandibular bone defect) classification, the treatment-oriented classification and treatment algorithm for CMFs.

## 2. Materials and Methods

### 2.1. Patients

Patients diagnosed as CMFs presenting to the Department of Oral and Craniomaxillofacial Surgery, Shanghai Ninth People’s Hospital affiliated to Shanghai Jiao Tong University School of Medicine, between January 2011 and January 2022, were involved in this retrospective study. Patients were divided into two groups, the treatment-algorithm-following group (after 2015) and treatment-algorithm-unfollowing group (before 2015), and retrospectively studied. All patients were treated by two qualified surgeons (Dr. Xu and Dr. Shi) at busy tertiary trauma institutions. A CT scan (CT, slice thickness, 1.25 mm, GE Healthcare, Buckinghamshire, England) was required for all patients before and 6 months after surgery. Edentulous patients or patients with systematic injuries were excluded from the study.

### 2.2. Ethical Statement

The study was conducted in accordance with the Declaration of Helsinki (as revised in 2013) and approved by the Shanghai Ninth People’s Hospital institutional review board (2016-161-T110, 17 October 2016). All patients who appeared in the figure gave full permission for their images and radiological documents to be used in publication. Signed informed consent forms were obtained from all patients.

### 2.3. Classification

All patients were classified based on the following four questions and then divided into five types (Figure S1):

1. whether a stable occlusal relationship could be regained (O);
2. whether a continuous mandibular morphology is reserved (C);
3. whether a high mandibular comminution is performed (C);
4. whether a segmental mandibular bone defect occurred (S).

**Figure 1:**
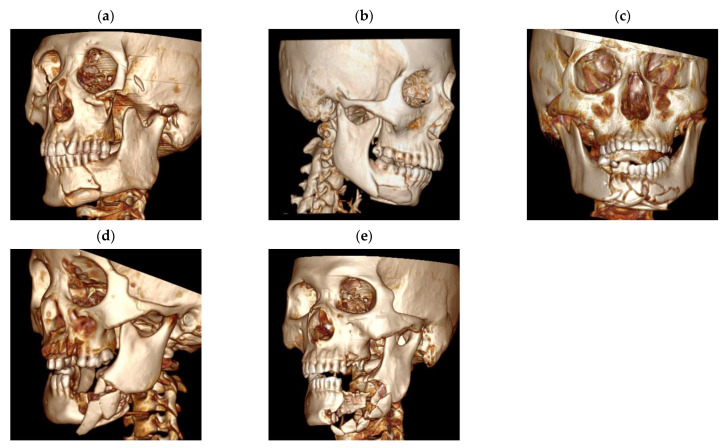
3DCT reconstruction of the OCCS classification of CMFs. (**a**) type I; (**b**) type II; (**c**) type III; (**d**) type IV; (**e**) type V; 3DCT: three-dimensional computed tomography; CMFs: comminuted mandibular fractures.

OCCS classification:

Type I: relatively intact occlusal relationship that can easily be reset and fixed by MMF, relatively sound mandibular morphology without any obvious mandibular continuity destruction or bone defect (Figure 1a);

Type II: relatively intact occlusal relationship, damaged mandibular morphology that broken into several large fracture fragments but it performs with a low degree of comminution and an intact mandibular continuity without segmental bone defect (Figure 1b);

Type III: mandibular morphology is damaged and broken into a mass of small comminuted bone fragments (even un-vascularized) with a higher comminution degree but the mandibular continuity is intact without segmental bone defect. The occlusal relationship can still be regained by MMF (Figure 1c);

Type IV: comparing to the type III fracture, it is unable to regain the occlusal relationship only by MMF and the mandibular continuity is impaired without segmental bone defect (Figure 1d);

Type V: segmental bone defect and mandibular continuity loss, poor occlusal relationship and damaged mandibular morphology (Figure 1e).

### 2.4. Management Algorithm

Most patients with type I fractures can be managed conservatively: for patients with a sound and stable occlusal relationship, no treatment is required; for patients with malocclusion, MMF with Winter’s dental arch bar and elastic traction for 3–4 weeks are performed because of no seriously displaced fragment.

For patients with type II, type III and type IV fractures, open reduction and internal fixation (ORIF) is necessary.

**Figure 2:**
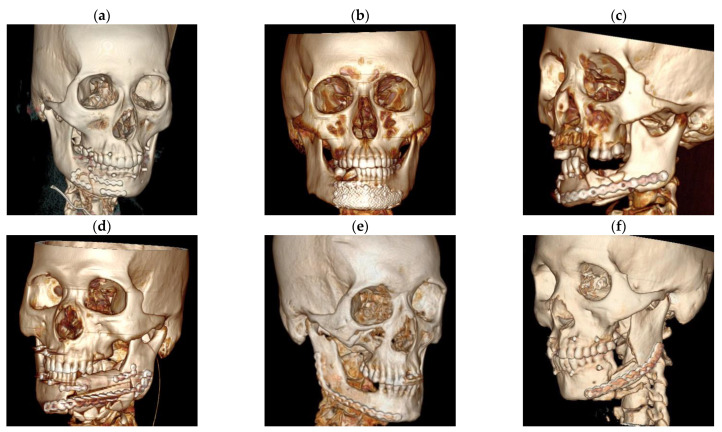
Postoperative 3DCT reconstruction of each fracture category. (**a**) Type II fracture managed with miniplates fixation following the Champy’s principle; (**b**) type III fracture, MMF, staged surgeries and titanium mesh fixation were performed; (**c**) type IV fracture managed with reconstruction plate systems fixation following the AO/ASIF principles; (**d**) reconstructed with fibular microvascular osteocutaneous free flap; (**e**) reconstructed with scapular microvascular osteocutaneous free flap; (**f**) reconstructed with iliac microvascular osteocutaneous free flap. 3DCT: three-dimensional computed tomography; MMF: maxillomandibular fixation; AO/ASIF: Arbeitsgemeinschaft fuer Osteosynthesefragen/Association for the Study of Internal Fixation.

In type II fractures, fixing fracture fragments with miniplates through an intraoral vestibular incision would be enough (the extraoral wound can also be used as an approach when it is big enough to access the fracture site) (Figure 2a).

In type III fractures, staged surgeries should be performed. Stage-one surgery should be performed 7 to 10 days ahead of ORIF, for debridement, hemostasis, restoration of the occlusal relationship through MMF (Figure S2a) and suture the wound, according to our previous experience [7]. If combined with other maxillofacial fractures, such as zygomatic-maxillary complex or condylar fracture, it is necessary to treat these fractures by ORIF during the stage-one surgery because it is helpful to obtain a favorable occlusal relationship. After 7–10 days, CMFs would be reduced in the stage-two surgery through an extra-oral approach according to the occlusal relationship and fixed with a pre-shaped 0.6 mm titanium mesh (Stryker, Kalamazoo, MI, USA) plus mini-plates when necessary (Figure 2b and Figure S2b). MMFs were removed 7–10 days after surgery.

For patients with type IV fractures, the classical AO/ASIF principles should be strictly followed, by ORIF with reconstruction plate systems through extra-oral incision to achieve osteosynthesis (Figure 2c).

For patients with type V fractures, microvascular osteocutaneous free flap transplantation is a good choice (Figure 2d–f). The donor sites include the fibula, iliac, and scapula.

During operations, MMF is needed for all patients intending to acquire a stable occlusal relationship before the rigid fixation. A strictly layered suture should be performed and antibiotics are required for three days after surgery.

### 2.5. Outcomes

Perioperative data including demographics, physical examinations, and other information were obtained from electronic medical records, including age, sex, systematic history, etiology, whether cases involved an isolated CMF or one combined with other maxillofacial fractures, the operation room time, length of stay in hospital, and whether a reoperation was performed (staged surgery was not considered as reoperation). All patients were visited at 1, 3 and 6 months after the surgeries.

Complications were recorded during follow-up visit, including infection, bone non-union, chin deviation, malocclusion, ipsilateral marginal mandibular nerve branch injury/weakness and limited mandible movement.

The Edward Ellis III method was used for the occlusal relationship assessment [8] while the evaluation timing was chosen as 1 month after discharge. Basically, three photographs were taken: frontal and right and left lateral. All were taken with the patient in maximum occlusion. One surgeon and one orthodontist independently examined each set of photographs and rated the occlusion as good (normal for the patient), poor (abnormal for the patient), or undecided (could not decide from photographs). The surgeon and the orthodontist were blinded to the treatment patients received.

MIO was defined as the vertical distance in millimeters between the incisal edges of the maxillary and mandibular central incisors, where they should be, or as medial to where they should be and measured with a ruler by one same doctor. Additionally, the mandibular functional assessment [9] was evaluated 1 month after discharge, and it was evaluated by two providers: protrusion (forward movement), retraction (backward movement), elevation (swing closed), and depression (swing open). A three-point functional scale was used (0 = no movement, 1 = limited movement, and 2 = normal movement).

Chin deviation was measured in degrees by the angle formed by the facial midline (the line connects glabella and nasi tip) and the straight line that connects the nasi tip and pogonion in the frontal photo. The degree of difference value preoperative and 1 month after discharge was employed for evaluation. The preoperative photo was obtained from the ID card. Over two degrees of change were defined as the chin deviation in this study.

### 2.6. Statistical Analysis

In the two-treatment group, continuous variables were presented as the mean ± standard deviations (range) and analysed by *t*-test or Mann–Whitney U test according to the normal distribution test. Chi-square or Pearson’s chi-square test, Continuity Correction, and Fisher’s Exact test were used to analyze clinical data. All hypothesis-generating tests were two-sided at a significance level of 0.05. Statistical analysis was performed using SPSS version 25.0 (SPSS Inc., Chicago, IL, USA).

## 3. Results

### 3.1. Demographics

A total 131 patients were included in this study; 22 were lost to follow-up visit. The remaining 109 patients (41 females and 68 males), ranging from 8 to 58 years of age, averaging 30.64 years, were included in the study. A total of 47 patients were treated unfollowing the treatment algorithm (admitted before 2015) and the other 62 were treated following the treatment (admitted after 2015). The causes of the fractures were high fall injury in 39 (35.78%), traffic accidents in 25 (22.93%), blunt injury in 31 (28.44%), stumble in 10 (9.17%), gunshot wounds in 2 (1.83%), and high-explosive injury in 2 (1.83%). In the following group, 26 (41.94%) patients had isolated CMFs, and in the unfollowing group, 22 (46.81%) patients had isolated CMFs. The mean operation room time for the following group was 186.55 min (the sum time were calculated for staged surgeries) and 153.19 min for unfollowing group. For the mean length of staying in hospital, the following group was 9.90 days and the unfollowing group was 10.63 days (Table 1).

### 3.2. Classification

According to CT scans, type I fracture (17.43%, [*n* = 19]), type II fracture (38.53%, [*n* = 42]), type III fracture (29.35%, [*n* = 32]), type IV fracture (11.01%, [*n* = 12]) and type V fracture (3.67%, [*n* = 4]) were found.

### 3.3. Outcomes

At least 6 months of follow-up visit (ranged from 6 months to 42 months, average 7.33 months) was required for all patients. Postoperative complications were observed in both groups. In the 62 patients of the algorithm-following group, 5 manifested infection, 4 manifested malocclusion, and 1 manifested bone non-union. A higher postoperative complication rate was observed in the algorithm-unfollowing group. In these 47 patients, 10 manifested infection, 5 manifested bone nonunion, and 3 manifested malocclusion (Table 2). The postoperative infection rate was found to be different and showed statistical significance between the two groups (χ^2^ = 3.932, *p* = 0.047). Only 2 patients experienced reoperation in the following group, but the counterpart in the unfollowing group was 8 and statistical significance was found (χ^2^ = 4.563, *p* = 0.033). The main reasons for them to experience reoperation were postoperative infection, bone non-union and secondary dento-maxillofacial deformities. Debridement, orthognathic surgery or microvascular osteocutaneous free flap transplantation was performed, accordingly (Table 3).

## 4. Discussion

The mandible is the most protruding bone and is susceptible to fracture in the maxillofacial region, with a fracture incidence rate of 23.8% to 81.3% [10,11,12], while CMFs account for only approximately 5–7% [2]. CMFs mainly result from high-energy impacts, directly or indirectly, to a localized area of the mandible [13,14] and may lead to a series of unfavorable consequences, such as airway obstruction, malocclusion, mouth-opening limitation, infection or bone nonunion, and secondary dento-maxillofacial deformities [2]. The treatment methods include MMF, external fixation [6], ORIF with micropletes [15], mini-plates, reconstruction plates [16] or titanium mesh [7]. ORIF with load-bearing reconstruction plates is advocated by AO/ASIF and is regarded as the first place internationally for the treatment of CMFs. Although these methods are all effective, it should attach to its own particular fracture category and consider comprehensively (the fracture orientation, amount of fracture fragments, displacement and blood supply protection) due to its complexity and the accompanying high postoperative complication rate because of the improper management, and thus, a personalized treatment plan should be attached to different patients (Figure S1)

After a life-threaten injury is managed, the top priority for CMF management is judging whether a segmental bone defect occurs. A microvascular osteocutaneous free flap transplantation (Figure 2d–f) should be performed to restore the continuity in this situation because of its strong resistance to infection and the ability of restoring the mandibular continuity and bone defect. Almost twenty years ago, fibular microvascular osteocutaneous free flap transplantation had already been reported in the reconstruction of severely comminuted atrophic mandible fracture [17]. Although bone tissue engineering (BTE) has been used in the reconstruction of a large post-traumatic mandibular defect, it still needs auto-graft, forearm flap, for vascularization [18]. Moreover, BTE may not be used at the very beginning after accident for the uncertainty of its survival rate due to the poor blood supply and high postoperative infection rate. Thus, microvascular osteocutaneous free flap transplantation and prophylactic tracheotomy are still necessary in the management of the type V, segmental bone defect CMFs (Figure 1e).

Before the widespread advocation of fixation systems by AO/ASIF, MMF had long been performed in the management of CMFs due to its low cost, easily handling, and no blood supply damage from stripping soft tissue. MMF is now only regarded as an adjunct to the fixation system [19] since the exclusive performance of MMF could lead to a series of complications [20,21]. However, under certain circumstances, when the mandibular morphology is sound with adequate osseous contact between the fragments and no mandibular continuity destruction, MMF is still effective and there is no need for ORIF. In other words, no open surgery is needed in the management of the type I fractures (Figure 1a) for a sound morphology with intact continuity, and slightly displaced bone fragments are stable enough and the blood supply could be better reserved to nourish and protect more bone tissue.

In cases of damaged mandibular morphologies without segmental bone defects or impaired mandibular continuity, the comminution degree may pose a significant effect on determining the treatment method. Damaged mandibular morphology and displaced bone fragments are indications of open surgery. In patients with a low or minimum degree of comminution, its blood supply is only slightly or partly damaged and the bone fragments are big enough to be fixed easily and immobilized with mini-plates following the Champy principles. It is similar to the management of single-line or multiple mandibular fracture, which fixing with mini-plates. It has been proposed that small and large titanium plates are equally effective for treating mandible fractures [22]. Even microplates with a three-level fixation technique are appropriate for the reconstruction of comminuted mandibular fractures without bony defects [15]. Both mini-plates and reconstruction plate systems used for internal fixation of mandible fracture are mostly successful in restoring functional occlusion. Only in bone fragments requiring the removal of over 1 cm can reconstruction plate systems achieve better outcomes because of its load-bearing characteristic [16]. Thus, mini-plate systems may be enough to fix type II fractures (Figure 1b), a damaged mandibular morphology with a low degree of comminution and an intact mandibular continuity.

When dealing with a higher comminution degree, the blood supply is often damaged. If the continuity of the mandible is intact and a stable occlusal relationship can be obtained based on the relatively intact dentition, MMFs in stage-one surgery should be performed to ensure a temporary normal occlusal relationship. Staged internal fixation has been recommended for the treatment of severely comminuted or infected mandible fractures to give enough time for the damaged periosteum, muscle, and mucosa to grow and to restore the vascular supply needed for bony healing [6], and thus, a greater amount of bone can be retained, and the second-stage ORIF can only be performed then. It should be fixed with titanium meshes [7] because of its malleability, extensive area coverage and high porosity. Though reconstruction plate systems may provide more load-bearing osteosynthesis, in this fracture category, titanium mesh is rigid enough because the mandibular continuity is intact. The malleability of it may facilitate accurate anatomical reduction for complete bony contact, and its extensive coverage can easily span the region of comminution, providing stability across the fracture site with small fragments. Moreover, the periosteal blood supply may nourish the bone segments through the perforations of it and promote bone healing [7] and reserve more bone tissues [23]. Thus, staged surgery is the best choice in the management of the type III fracture (Figure 1c).

In terms of the type IV fracture (Figure 1d), for CMFs with severely impaired dentition, in which a stable occlusal relationship cannot be easily pieced and fixed with only MMF and the mandibular continuity is impaired, the classical AO/ASIF principles should be followed. A load-bearing osteosynthesis appliance, the reconstruction plate systems, can totally bear a functional load from the bite force and avoid relative motion [2] and, as a consequence, ameliorate the postoperative complication rate.

The main reason for patients to experience reoperations is the improper fixation method and timing. Before the year 2015, we used to struggle in the practice of load-bearing and load-sharing systems, and the improper performance of the load-sharing system with mini-plates should be blamed for the postoperative infection, secondary dento-maxillofacial deformities and bone non-union. In the algorithm-unfollowing group (before year 2015), some patients received premature open surgery, which exacerbates the already damaged vascular supply to the fracture site. So, the best choice in the circumstance is to give enough time for the periosteum, muscle and mucosa to restore vascular supply needed for bony healing and perform ORIF later [6]. Massive soft-tissue defects represent another important factor leading to postoperative infection. However, not many patients with severe soft tissue defects were encountered for the strict control of firearms and explosives by the Chinese government. Based on previous experience, in patients with severe soft tissue defects or penetrating wounds, it is one of the key factors to gain a good seal from intraoral mucosa to extraoral skin. A patient was found to be administered a tongue flap for sealing the wound when referred to our hospital, and he gained quite a good therapeutic effect afterward.

This study still has its limitations. First, a better occlusal relationship assessment should be applied. For the poor MIO and compliance of some patients before surgery, it is inconvenient to obtain an objective grading system (OGS) or peer-assessment rating (PAR) index. The T-scanner was not included because it was not used to all patients for the long time span of the study; therefore, no complete data were available. Second, FACE-Q scales [8], a validated patient-reported outcome measure used to measure patient satisfaction with facial aesthetics, was not included for the long time span of the study. Third, as this study had a small sample size, a single-center retrospective study and large-sample-size, multi-center prospective clinical study with the aforementioned scales would be performed in the future. Edentulous patients were excluded from the study for the small sample size of the patient group and its unique characteristics in treatment, especially atrophic mandibles, such as extremely poor blood supply, being prone to bone non-union, and usually poor systematic conditions, etc., and should thus be sorted into a new type of CMF. It is worth noting that sometimes difficult-to-define fractures or misjudgment before the surgery can occur and a more appropriate treatment method should be applied without hesitation. If always stereotyping to the classification, the function or morphology will not be guaranteed, which will affect the outcomes.

## 5. Conclusions

In CMF management, reconstruction plate systems may not always be the best choice and are unsuitable for covering all the categories in CMFs in the consideration of maximum function and morphology recovery and minimizing the potential iatrogenic trauma to patients. In this study, according to the occlusal relationship, comminution degree, mandibular continuity, and whether segmental bone defects are present, a treatment-oriented classification for CMFs, OCCS classification, is created, which is able to ameliorate the complication rate and improve outcomes.

## Figures and Tables

**Table 1:** CMFs Groups: treatment algorithm following versus unfollowing.

Characteristic *	Following	Unfollowing	*p*
Age, yr	29.58 ± 10.11 (12, 51)	32.04 ± 10.68 (8, 58)	0.873
Gender			0.503
Female	25, 40.32%	16, 34.04%	
Male	37, 58.68%	31, 65.96%	
Comorbidity	32, 51.61%	26, 55.32%	0.701
Mechanism			0.528
Fall	23, 37.10%	16, 34.04%	
TA	15, 24.19%	10, 21.28%	
BI	14, 22.58%	17, 36.17%	
Stumble	6, 9.68%	4, 8.51%	
GSW	2, 3.22%	0, 0.00%	
HEI	2, 3.22%	0, 0.00%	
Isolated vs. additional CMFs	26, 41.94%	22, 46.81%	0.612
OR time, min	186.55 ± 87.09 (60, 480)	153.19 ± 69.72 (60, 300)	0.159
LOS, days	9.90 ± 3.04 (4, 20)	10.63 ± 3.31 (5, 18)	0.254

CMFs: comminuted mandibular fractures; TA, traffic accident; BI, blunt injury; GSW, gunshot wound; HEI, high-explosive injury; OR, operation room; LOS, length of stay. * Patient demographic and preoperative data of the two groups showed similarities in age, gender, comorbidity, mechanism and simultaneous CMFs and no statistical significance was found between the two groups in terms of the classification. OR time for type III fracture is the sum of the two surgeries.

**Table 2:** Complications of the Two Groups.

Complication	Following	Unfollowing	*p*
Infection *	5, 8.06%	10, 21.28%	0.047
Bone non-union	1, 1.61%	5, 10.64%	0.105
chin deviation	5, 8.06%	6, 12.77%	0.627
Malocclusion	4, 4.84%	3, 6.38%	1.000
Numb	41, 66.13%	32, 68.09%	0.830
Reoperation *	2, 3.23%	8, 17.02%	0.033
MIO change, mm	13.02 ± 3.52 (5, 20)	12.55 ± 3.69 (5, 20)	0.507
Functional score (0–2)	1.71	1.74	0.269

MIO, maximal interincisor opening; MIO and Functional score was measured before the operation and 1 month after discharge. Numb was counted when reported by patients for over 3 months. * Statistical significance between the algorithm-following and algorithm-unfollowing groups (*p* < 0.05).

**Table 3:** Patients experienced reoperations.

Category	Algorithm	Time of Re-Op	Interval *, mo	Cause	Operation
patient 6	Un-Fo	1	0.75	If	De
patient 11	Un-Fo	1	1.5	BNU	De
patient 15	Un-Fo	2	2	BNU	De, MOFT
patient 22	Un-Fo	1	6	MO, CD	OGS
patient 26	Un-Fo	1	2	BNU	De
patient 33	Un-Fo	1	4	MO, CD	OGS
patient 34	Un-Fo	2	1	BNU	De, MOFT
patient 42	Un-Fo	2	1	If, MO, CD	De, OGS
patient 57	Fo	1	2	BNU	De
patient 83	Fo	1	2	If	De

Re-Op: Reoperation; Un-Fo: Unfollowing; Fo: Following; If: Infection; BNU: Bone non-union; MO: Malocclusion; CD: chin deviation; De: Debridement; MOFT: Microvascular osteocutaneous free flap transplantation; OGS: Orthognathic surgery; * the interval between the first discharge and the first time of the reoperation.

## Data Availability

The data that support the findings of this study are available from the corresponding author upon reasonable request.

## Supplementary Materials

The following supporting information can be downloaded at: https://www.mdpi.com/article/10.3390/jcm11216301/s1, Figure S1: an overview of the classification of CMFs; Figure S2: a: the intraoral photo after stage one surgery of the type III fracture, b: the intraoperative photo of the type III fracture.
