# Supplementary material for: OCCS Classification and Treatment Algorithm for Comminuted Mandibular Fractures Based on 109 Patients and 11 Years Experiences: A Retrospective Study
Source: J Clin Med. 2022 Oct 26;11(21):6301. doi: 10.3390/jcm11216301 (PMC9657356; doi:10.3390/jcm11216301)
Supplement: Supplementary file 1 [file jcm-11-06301-s001.zip › jcm-1965965-supplementary.pdf]

Supplementary Materials:

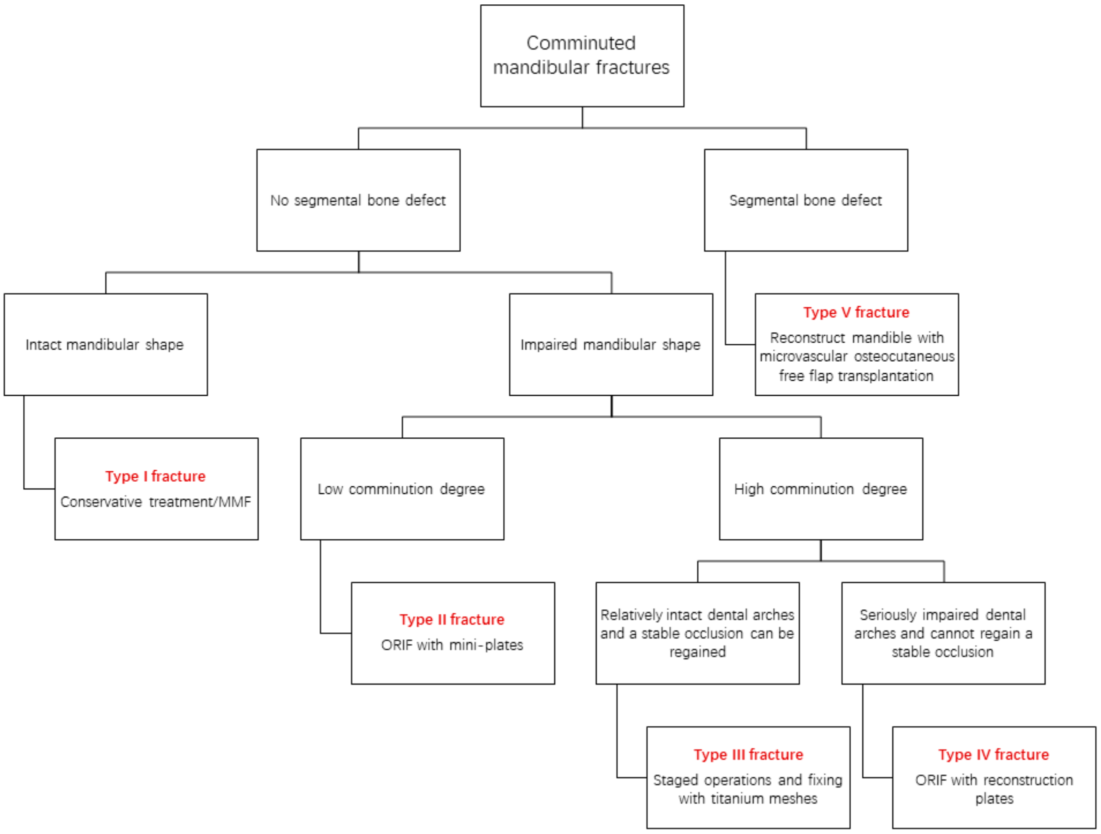

Figure S1. mind map of the OCCS classification for comminuted mandibular fractures.

a	b
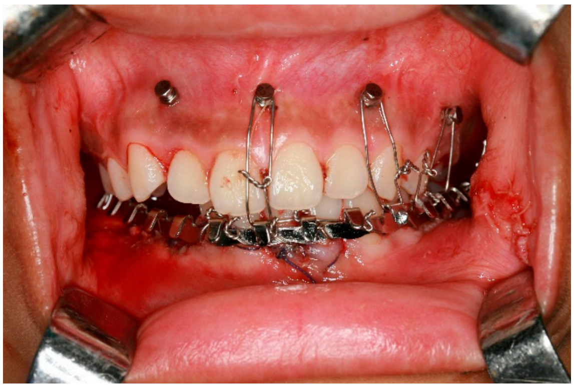	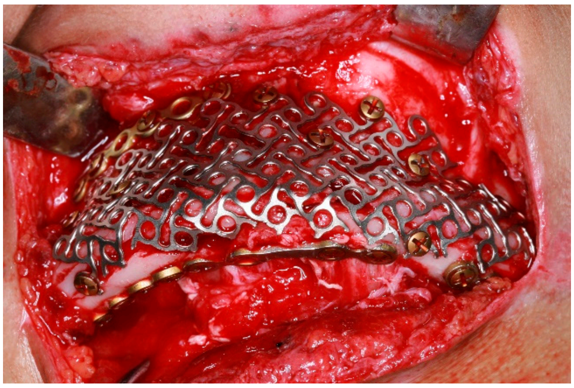

Figure S2. photographs of the type III fracture. (a) after stage one surgery, wound was sutured and the occlusal relationship was restored and fixed with MMF; (b) the intraoperative photograph, titanium mesh and mini-plates were used to fix the fracture site. MMF: maxillomandibular fixation.
